# The Veterinary Association of Manitoba

**Published:** 1901-03

**Authors:** F. Torrance

**Affiliations:** Secretary


Secretary.

### THE VETERINARY ASSOCIATION OF MANITOBA.

The annual meeting was held in the City Hall, Winnipeg, on February 19, 1901, the President, Mr. J. G. Rutherford, in the chair. The following members were present: J. G. Rutherford, S. A. Coxe, W. E. Martin, J. Welch, W. J. Hinman, J. J. Irvine, J. G. Cruikshank, R. E. Monteith, A. E. Williamson, H. J. Johnston, W. A. Hilliard, W. A. Dunbar, J. A. Stevenson, W. R. Taylor, J. W. Routledge, J. D. McGillivary, G. Hilton, W. Swenerton, J. H. Lipsett, J. Golley, W. S. Henderson, H. J. Elliott, C. Little, H. D. Smith, and W. H. Smith.

After routine the report of the Secretary-Treasurer and Registrar was presented, showing the Association to be in a flourishing condition. During the year the membership has increased to a total of 71. The finances were in a satisfactory condition, showing a balance of $461.91 to the credit of the Association. The auditors, Messrs. Little and H. D. Smith, reported having examined the books and vouchers and found everything correct. The reports were adopted.

The meeting then proceeded to the election of officers, as follows: President, W. A. Dunbar, Winnipeg; Vice-President, S. A. Coxe, Brandon; Secretary-Treasurer and Registrar, F. Torrance, Winnipeg. Examiners—W. A. Dunbar, W. E. Martin, and F. Torrance. Council—W. Swenerton, J. G. Rutherford, W. E. Martin, and W. H. Smith.

The President reported an interesting case of injury to the flexor tendons of the hind leg. Both perforans and perforatus tendons had been cut through in a runaway accident, and some time elapsed before he was called in, another practitioner having been first in attendance. The leg was found enveloped in a plaster-of-Paris bandage, and when this was removed the wound was discovered to be in a septic condition, with sloughing edges. Fever was high and the animal suffered greatly. A more rational treatment was adopted, consisting of placing the parts at rest by means of a very ingenious splint devised for the case by the doctor and by the application of antiseptic dressings. Under this treatment the horse had steadily progressed to recovery and was now able to resume his severe work of galloping to fires and trotting from them. In the discussion which ensued many members took part and Mr. Dunbar was warmly congratulated on the ingenuity of his splint and the success of his treatment.

Mr. Rutherford then presented a paper on "Intestinal Lesions in the Horse." The great experience of the essayist enabled him to deal with this subject from a practical rather than a theoretical stand-point, and the members present enjoyed a treat in listening to his paper. It led to an animated discussion in which many took part, and several curious and instructive experiences were related.

Dr. Elliott, of Brandon, followed with a paper on "Influenza in Dogs," giving his experience in a recent outbreak of the disease in Brandon and detailing the treatment which he had found most successful. In the discussion following attention was called to the frequency of strychnine poisoning in dogs and the best mode of treating it. In the opinion of the meeting nothing better than chloral hydrate was known.

Mr. Stevenson asked Dr. Torrance to give some account of the research into the pathology of "swamp fever" which Dr. Bell and he had undertaken.

In reply Dr. Torrance said that he hoped at a future date, when the investigation had reached more definite results, to make a written report on the subject. At present he would refer only briefly to the work that had been done. A small sum of money had been granted by the government for this research, and they had purchased two horses, upon which they had made experiments by inoculating them with pure cultures of the large bacillus which had been discovered in several cases of this so-called swamp fever. In one horse they had been partially successful in producing a modified form of the disease, but the other had proved refractory. This might have been owing to natural immunity or to attenuation of the virus from artificial cultivation. They had also made several post-mortem examinations, had made temperature-charts of cases for long periods of time, and had made numerous examinations of blood as well as blood-counts. In conclusion, he pointed out the importance of continuing the investigation into a disease which is probably the greatest menace to horse owners in this province, and asked the coöperation of the members in securing a further grant for this object and in contributing their experience with the disease.

Several members spoke on the subject and all agreed as to the importance of the investigation. It was moved by Mr. C. Little, seconded by Mr. Stevenson, and carried unanimously, that this Association petition the government to make a further grant to Drs. Bell and Torrance for the purpose of continuing their research into swamp fever in horses.

It was moved by Mr. Coxe, and seconded by Mr. Martin, that the sum of fifty dollars be given to Dr. Torrance for his services in this research; carried.

On motion of Dr. Hilliard, seconded by Mr. Stevenson, it was decided to hold the semi-annual meeting in Brandon, the date to be fixed by the Council.

Votes of thanks were passed to Dr. Bell and Dr. Torrance for their investigation, to the essayists for their valuable contributions, and to the City Council for the use of the room as a place of meeting. The meeting then adjourned.

F. TORRANCE,
Secretary.

